# Sexual dimorphism of leptin and adiposity in children between 0 and 10 years: a systematic review and meta-analysis

**DOI:** 10.1186/s13293-022-00454-y

**Published:** 2022-09-05

**Authors:** Jose Guillermo Ortega-Avila, Harry García-Muñoz, Alejandro Segura Ordoñez, Blanca C. Salazar Contreras

**Affiliations:** 1 Grupo de Investigación de Ciencias Básicas y Clínicas de la Salud, Departamento de Ciencias Básicas de la Salud, Pontificia Universidad Javeriana, Seccional-Cali, Cali, Colombia; 2 Grupo de investigación Salud y Movimiento, Facultad de Salud, Universidad Santiago de Cali, Cali, Colombia; 3 Grupo de Nutrición, Departamento de Ciencias Fisiológicas, Facultad de Salud, Universidad del Valle, Cali, Colombia; 4 Programa de Medicina, Facultad de Salud, Universidad Icesi, Cali, Colombia

**Keywords:** Leptin, Adiposity, Children, Sex, Normal-weight

## Abstract

**Background:**

Differences in adolescents and adults by sex in blood levels of leptin and adiposity have been described; however, it is not yet clear if these differences arise from the prepubertal stage in subjects with a normal-weight. Therefore, we examine whether there are differences by sex in levels of blood leptin and adiposity in children with a normal-weight between 0 and 10 years old.

**Methods:**

Search strategy: eligible studies were obtained from three electronic databases (Ovid, Embase and LILACS) and contact with experts. Selection criteria: healthy children up to 10 years of age with normal-weight according to age.

Data collection and analyses: data were extracted by four independent reviewers using a predesigned data collection form. For the analysis, we stratified according to age groups (newborns, 0.25–0.5 years, 3–5.9 years, 6–7.9 years, 8–10 years). The statistical analysis was performed in the R program.

**Results:**

Of the initially identified 13,712 records, 21 were selected in the systematic review and meta-analysis. The sex was associated with the overall effect on blood leptin (pooled MD = 1.72 ng/mL, 95% CI: 1.25–2.19) and body fat percentage (pooled MD = 3.43%, 95% CI: 2.53–4.33), being both higher in girls. This finding was consistent in the majority of age groups.

**Conclusion:**

The results of our meta-analyses support the sexual dimorphism in circulating blood leptin and body fat percentage between girls and boys with normal-weight from prepuberty.

**Supplementary Information:**

The online version contains supplementary material available at 10.1186/s13293-022-00454-y.

## Highlights

- Differences by sex in blood leptin and body fat percentage in children are present before 10 years old
- Girls have higher circulating leptin and body fat percentage than boys
- The sex differences in blood leptin and adiposity in children increase progressively with the age

**Supplementary Information:**

The online version contains supplementary material available at 10.1186/s13293-022-00454-y.

## Background

Leptin is a peptide hormone produced primarily by the subcutaneous white adipocytes, whose main functions are to regulate satiety and caloric intake [1]. The central action of leptin in hypothalamic neurons leads to reduced caloric intake and increases energetic expenditure in the long-term [2]. Additionally, leptin has been involved in the regulation of multiple processes such as immune response, endothelial function, and platelet activation among others [3]. The dysregulation in the synthesis and/or sensibility of this adipokine in both sexes has been associated during adulthood with the development of chronic diseases [4].

Levels of circulating leptin in women are higher than in men. Apparently, the main determinant of its concentration in blood is the fat mass, with which it has a strong statistical correlation [5–7]. Currently is assumed that the differences of both body fat and blood leptin levels rise during puberty and therefore these sexual dimorphisms may be associated with the changes in the levels of steroid sexual hormones [8–10]. However, some studies have suggested that the differences in the indicators of adiposity arise before adolescence [11]. Besides this controversy, the majority of the studies of leptin in children have been performed in populations with overweight and obesity where the sex influence usually is not analysed.

Studying the blood leptin levels and adiposity in prepubescent children with normal-weight by sex can help to give clarity on how these factors contribute to disorders in the body composition and metabolism during adolescence and adulthood [12–14]. Therefore, the aim of this systematic review/meta-analysis was to examine whether there are differences by sex in levels of blood leptin and body fat percentage in children with a normal-weight between 0 and 10 years old.

## Main text

This systematic review was conducted using a protocol following the guideline of Preferred Reporting Items for Systematic Review and Meta-Analyses (PRISMA) [15]. This protocol was registered in PROSPERO (code: CRD42020158478).

### Search strategy and inclusion/exclusion criteria

A search algorithm was applied to each of three electronic databases (Ovid, Embase and LILACS), without language or publication date restrictions, until June 2022. The terms used for the search were; "leptin", adiposity", "obesity", "body composition", "abdominal obesity", "pediatric obesity", "body adiposity index", "bmi trajectory", "body mass index", "waist circumference", "fat mass", "visceral adiposity index", "visceral fat", "fat thickness", "body fat percentage", "anthropometric indices", "body shape index", "anthropometric parameter", "obesity indices", "triceps skinfold thickness", "infant, "child", "children", and "adolescent" (Additional file 1). Search strategies were developed using text words as well as medical subject headings (MeSH) associated with leptin and body fat percentage in normal-weight children. While the exact terms varied somewhat depending on the database searched, keywords included such terms as "leptin", "body composition", "children", and "body fat percentage". To find studies, we also contacted experts in the field by email who were selected from the articles reviewed.

In order to reduce the effects of hormonal changes on results, we included observational studies and controlled clinical trials that included healthy children up to 10 years of age [16], with normal-weight (BMI ± 1 SD according to age) and simultaneous reports for leptin and body fat percentage discriminated by sex. Case studies, conference abstracts, letters to the editor, review articles, and articles without complete information or no response from the authors after contact by email were excluded.

### Data extraction and risk of bias assessment

A data registry format was tested with 40 articles (10 per reviewer), in which study identification information was filled and eligibility criteria were sought; the results were evaluated by a peer reviewer and later, as a group, possible discrepancies were discussed to reach consensus on the information to be contained in each cell. The results of the databases were included in a spreadsheet and duplicates were eliminated; four reviewers in pairs performed the selection of studies by title and abstract according to eligibility criteria and discrepancies were resolved by a third researcher from an alternative pair. The selected studies were reviewed taking into account the full text of each article by eligibility criteria; from these articles, the information on the variables was extracted.

We used the Newcastle–Ottawa Quality Assessment Scale as a tool of quality assessment for non-randomised studies [17], this scale uses a score to judge a study based on three broad perspectives: the selection of the study groups; the comparability of the groups; and the ascertainment of either the exposure or outcome of interest for case–control or cohort studies, respectively. To evaluate the quality of cross-sectional studies selected, we adapted the previously validated Newcastle–Ottawa Quality Scale for cohort studies [18], according to this scale, the studies were categorised into high-quality or low-quality studies using a score of 6 as a cut-off point.

### Statistical analysis

To estimate the effect of sex on blood leptin and adiposity in children between 0 and 10 years, the leptin concentrations were expressed in ng/mL when necessary. For the studies that reported medians and interquartile ranges, the mean and standard deviation (SD) were estimated using the methodology of McGrath et al. [19], if this was not the case, SD was estimated by standard equations from the standard error (SE) or confidence interval (CI) [20], if data were still insufficient for mean and SD estimation, an email request was sent to the authors.

We stratified the meta-analysis by age groups (Newborns, 0.25–0.5, 3–5.9, 6–7.9, 8–10 years). The effect size of weighted mean difference (MD) was estimated with a random-effect model since this model could incorporate the heterogeneity, and therefore proved a more generalised result. The heterogeneity of the average effect size was evaluated based on the calculation of the I2 index; if the value of this statistic was ≥ 75%, we considered it a high variation. We performed a sensitivity analysis using the leave-one-out method, iteratively excluding one study at each analysis, to assess the effect of one particular study on the pooled outcomes and confirm that our findings were not driven by any single study [21]. Outlier analysis was used to identify and estimate the effect of reports with extreme effect sizes in each age subgroup. Outlier studies were defined when the 95% CI was outside the 95% CI of the pooled effect. Subgroup analyses were run to find the source of heterogeneity considering, study design, technique of measurement, geographic region, and study quality. The publication bias was assessed by funnel plots and Egger's regression when the number of studies was ≥ 6 [22, 23].

The analysis was performed with the R programming language with R-studio platform version 4.0.2 (R Project for Statistical Computing, https://www.r-project.org/) using Meta, Estmeansd, and Dmetar packages [24, 25]. A *P*-value ≤ 0.05 was considered statistically significant.

## Results

### Study identification and selection

**Fig. 1:**
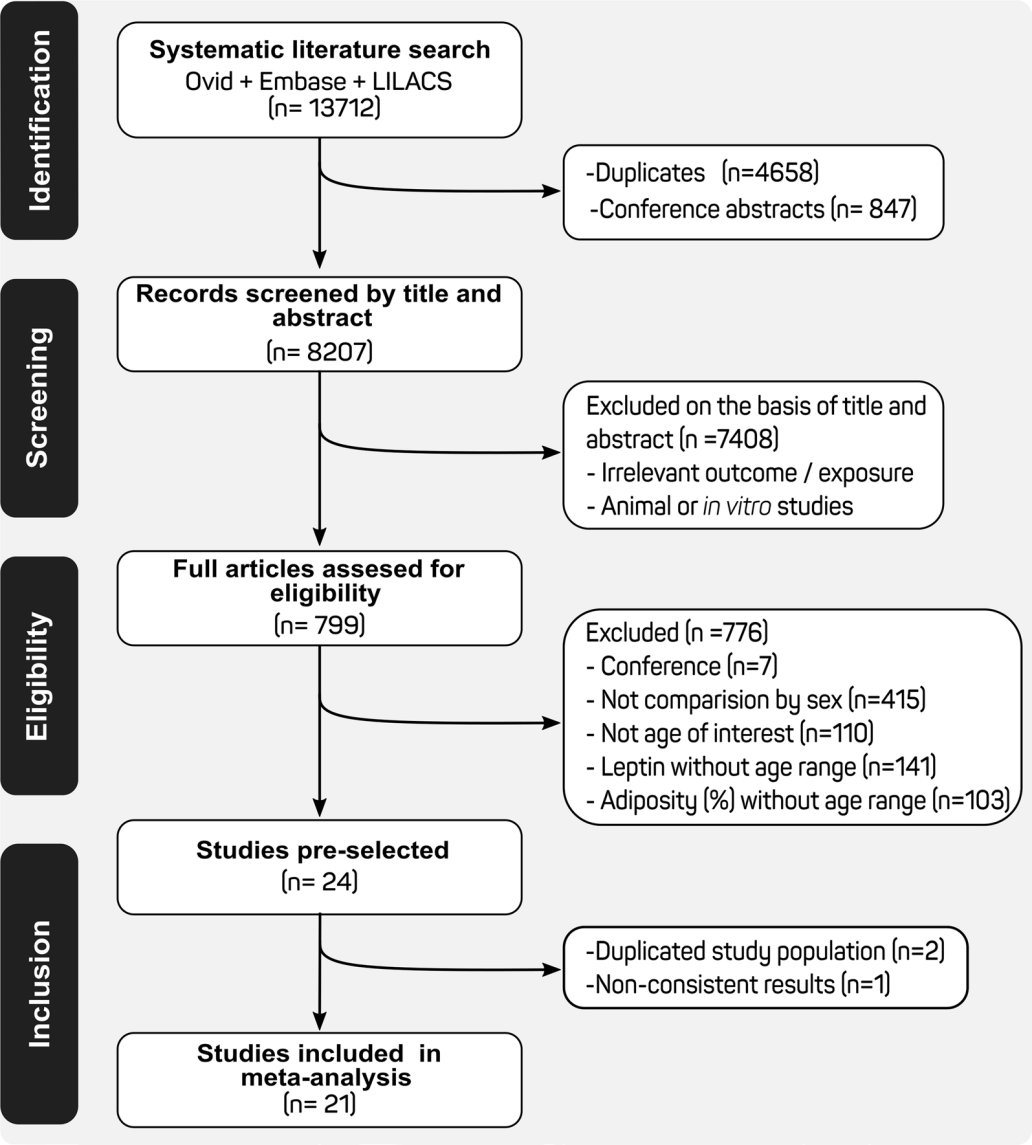
Systematic review flow chart

A total of 13,712 articles were identified in an initial search. After eliminating 4658 duplicate studies and 847 reports of conferences, 8207 were screened by title and abstract, and 7408 were eliminated because not meet the eligibility criteria. A total of 799 articles were reviewed in full text. Of those, 22 articles met our inclusion criteria; one article was excluded because the mean and SD of body fat percentage were considered biologically not plausible [26]. Finally, 21 articles were included in the systematic review and meta-analysis [27–47] (Fig. 1).

### Characteristic of selected studies

In the 21 studies selected (Table 1), a total of 5619 girls and 5692 boys were included for leptin, while 5758 girls and 5870 boys for body fat percentage. The articles were published in English language between 1999 and 2022, from Europe (*n* = 10), America (*n* = 5), Oceania (*n* = 3), Asia (*n* = 3). Sample sizes ranged from 12 to 4633 subjects; ten studies had a total sample size greater than 200 subjects. Study designs were as follows; cross-sectional (*n* = 12), and cohort (*n* = 9). The blood leptin was assessed in plasma (*n* = 8) or serum (*n* = 11); two studies did not report the type of blood sample used [33, 34]. The majority of studies reported that blood samples were drawn after fasting, except the report of Dencker et al. [44], and those studies that included umbilical cord blood samples [27–29]. Concentrations of leptin were determined by enzyme-linked immunosorbent assay (ELISA) (*n* = 5), radio immuno assay (RIA) (*n* = 14), and Milliplex multiplex assays (MMA) (*n* = 2).

**Table 1:** Characteristic of studies selected

Studies by age groups	Study design	Country	Sample size by sex		Age (years)		BMI/BMI z, score/BMI sds/ Weight		Body fat percentage			Circulating leptin			
			Girls *n* (%)	Boys *n* (%)	Girls mean(± SE)	Boys mean(± SE)	Girls	Boys	Girls means (± SE)	Boys means (± SE)	Method	Girls (ng/mL)	Boys (ng/mL)	Blood sample	Method
Newborns															
Okereke [27]	Cross-sectional	EEUU	32 (41)	46 (59)	–	–	3.2 (0. 41)^α^	3.5 (0.5)^α^	11.25 (3.98)	11.9 (4.4)	TOBEC	16 (13.8)	12.7 (12.8)	Serum	RIA
Javaid^)^ [28]	Cohort	UK	50 (43)	67 (57)	–	–	3.2 (0.5)^α^	3.51 (0.5)^α^	15.9 (13.6;19.4)^¶^	14.5 (12;16)^¶^	DXA	9.1(5.9;13.9)^¶^	7.4 (4.2;11.4)^¶^	Serum	RIA
Euclydes [29]	Cohort	Brazil	62 (59.6)	42 (40.4)	–	–	3.4 (0.4)^α^	3.34 (0.4)^α^	9.8 (4.1)	8.2 (3.6)	PG	29 (26.9)	22.8 (25.1)	Plasma	ELISA
0.25 to 0.5 years															
Estampador [30]	Cohort	Sweden	15 (48)	16 (52)	0.32 (0.03)	0.32(0.03)	6.2 (1.07)^α^	6.9 (0.69)^α^	25.6 (0.79)	27.1(0.25)	PG	4.9 (2.9)	4 (1.6)	Plasma	RIA
de Fluiter [31]	Cohort	Netherland	138 (46)	159 (54)	0.25	0.25	5.7 (5.1;6.2)^α^	6.2 (5.7;6.6)^α^	23.3 (19.6;26.5)^¶^	22.1(19.0;24.6)^¶^	PG	1.7(1.5;1.9)^¶^	1.3 (1.1;1.5) ^¶^	Plasma	MMA
			138 (46)	159 (54)	0.5	0.5	7.3 (6.8–7.8)	7.9 (7.3;8.4)	25.1 (21.6;28.9)^¶^	23.5 (20.0;26.7)^¶^	PG	0.9(0.6;1.1)^¶^	0.8 (0.7;1.0) ^¶^	Plasma	MMA
3 to 5.9 years															
Erhardt [32]	Cohort	Europe (eight countries)	47 (53)	42 (47)	3 to 3.9^❢^	3;3.9^❢^	16.2 (1)	16.3 (0.9)	16.8 (2.9)	15.6 (2.5)	Skinfolds	2.0 (1.5;3.8)^¶^	1.5 (1.1;2.5) ^¶^	Serum	ELISA
			33 (50)	33 (50)	4 to 4.9^❢^	4;4.9^❢^	15.8 (0.8)	16 (0.8)	16.6 (2.5)	15.1 (2.3)	Skinfolds	1.9 (1.5;2.7)^¶^	1.3 (1.0;1.7) ^¶^	Serum	ELISA
			32 (53)	28 (47)	5 to 5.9^❢^	5;5.9^❢^	15.6 (0.9)	15.7 (0.9)	16.5 (3.2)	14.5 (3.2)	Skinfolds	1.9 (1.5;2.7)^¶^	1.2 (0.9;2.4) ^¶^	Serum	ELISA
Jáuregui [33]	Cross-sectional	Mexico	197 (49)	203 (51)	4.7 (0.5)	4.8(0.6)	0.2(1) ^†^	0.2 (1.1) ^†^	25 (5.7)	22.8 (5.8)	BIA	3.5 (3.1)	2.6 (2.7)	-	ELISA
Francis [34]	Cohort	EEUU	365 (48)	396 (52)	4.8 (0.71)	4.8(069	0.05(0.9)	0.2(1.1)	19.7(6.8)	19.9(6.6)	PGA	6.7 (1.9)	3.83(1.0)	-	MMA
6 to 7.9 years															
Garnett [35]	Cohort	Australia	137 (54)	118 (46)	7.8 (0.6)	7.9(0.6)	16.9 (2.3)	16.7 (2.5)	23.6 (8.4)	17.4 (8.6)	DXA	3.4 (3.2;3.6)^❡^	2.2 (2.0;2.4)^❡^	Serum	RIA
Kim (2011) [36]	Cross-sectional	Korea	229 (50)	231 (50)	7.8 (0.5)	7.9 (0.5)	15.5 (1.4)	16 (1.2)	16.7 (4.1)	13.8 (4.1)	BIA	3.4 (2.1)	2.5 (1.8)	Serum	RIA
Metcalf [37]	Cohort	UK	158 (56)	122 (44)	6.9 (0.3)	6.9 (0.3)	16.4 (2.8)	15.7 (2.1)	21.9 (12.8) ^ℑ^	14.4 (8.0) ^ℑ^	DXA	4 (5.2) ^ℑ^	2.6 (2.7) ^ℑ^	Serum	RIA
			148 (55)	121 (45)	7.8(0.3)	7.9 (0.3)	16.9 (3.1)	15.9 (2.3)	23.3 (14.5) ^ℑ^	14.8 (9.8) ^ℑ^	DXA	4.4 (6.6) ^ℑ^	2.6 (3.1) ^ℑ^	Serum	RIA
Jeffery [38]	Cohort	UK	101	134	7.0	7.0	0.54 (0.22) ^‡^	0.21 (0.18) ^‡^	30 (1.72) ^§^	26 (1.2) ^§^	Skinfolds	4.6 (1.3) ^§^	2.7 (0.6) ^§^	Serum	RIA
Erhardt [32]	Cohort	Europe (eight countries)	41 (48)	45 (52)	6 to 6.9^❢^	6; 6.9^❢^	15.8(1.1)	15.8 (1)	16.9 (2.7)	13.6 (2.9)	Skinfolds	2.7 (1.5;3.7) ^¶^	1.3 (0.8;2.5)^¶^	Serum	ELISA
			72 (53)	65 (47)	7 to 7.9^❢^	7; 7.9^❢^	15.9(1)	15.9 (1.2)	16.5 (3.1)	14.5 (3.5)	Skinfolds	2.0(1.4;3.8)^¶^	1.7 (1.0;3.3)^¶^	Serum	ELISA
Vitery [39]	Cross-sectional	Colombia	56 (51)	54 (49)	7.9 (1.2)	7.8 (1.3)	16.4(1.8)	16.1 (1.2)	22.4 (4.1)	18.4 (3.5)	Skinfolds	6.9 (5.0)	3.3 (3.7)	Plasma	ELISA
Haapala [40]	Cohort	Finland	192 (49)	198 (51)	7.6 (0.4)	7.7 (0.4)	− 0.2(1.1) ^†^	− 0.2 (1.1) ^†^	22.2 (7.4)	17.2 (7.7)	DXA	5.9 (4.2)	4.2 (3)	Plasma	RIA
8 to 10 years															
Byrnes [41]	Cross-sectional	Australia	30(51)	29(49)	8.6 (0.2)	8. 5(0.3)	0.5^†^	0.3^†^	25.9 (1.1)	18.4 (1.2) ^§^	BIA	11.5 (2.2) ^§^	6.5 (1) ^§^	Plasma	RIA
Arrowsmith [42]	Cohort	Australia	12(46)	14(54)	7.9 (0.8)	8.3 (0.8)	16.6 (2.2)	18.3 (3.7)	19.9 (4.5)	19.2 (5.8)	Skinfolds	6.2 (3.7)	8.3 (5.6)	Plasma	RIA
Celi[43]	Cohort	Italy	395	447	8.5(7.2;9.6)^❡^	9.4(8.3;10.6)^❡^	20.6 (16.1;24.1)	21.5(16.1;24.8)	35.2 (11.1)	31.7 (11.7)	Skinfolds	11.9(5.2;20.6)^❡^	9.9(3.8;18.4)^❡^	Serum	RIA
Dencker [44]	Cross-sectional	Sweden	79(46)	91(54)	9.8 (0.6)	10 (0.6)	17.4 (2.7)	17.6 (2.6)	21.9 (8.4)	16.1 (8.3)	DXA	5.3 (4.8)	3.2 (4.2)	Serum	RIA
Yamborisut [45]	Cross-sectional	Thailand	28 (55)	23 (45)	8.1 (0.9)	8.3 (0.9)	− 0.11^†^	− 0.35^†^	16.9 (5.6)	12.6 (4.7)	BIA	6 (2.6)	3.7 (2.4)	Serum	RIA
Metcalf [37]	Cohort	UK	148 (56)	114 (44)	8.9 (0.3)	8.9(0.3)	17.9 (3.7)	16.4 (2.8)	26.4 (13.4) ^ℑ^	16.6 (12.7) ^ℑ^	DXA	6.7 (7.1)^ℑ^	3.8 (3.8)^ℑ^	Serum	RIA
			147 (56)	115 (44)	9.9 (0.3)	9.9 (0.3)	18.4 (5)	17 (3.1)	26.8 (14)^ℑ^	18.9 (15.3) ^ℑ^	DXA	8.2 (12.7)^ℑ^	4.2 (6.1)^ℑ^	Serum	RIA
Jeffery [38]	Cohort	UK	101 (43)	134 (57)	8.0	8.0	0.53^‡^	0.28^‡^	30.1 (2.1) ^§^	26.6 (1.7) ^§^	Skinfolds	5.3 (1.7)	3.2 (1.2)	Serum	RIA
			101 (49)	134 (51)	9.0	9.0	0.61^‡^	0.38^‡^	32.3 (1.8) ^§^	27.8 (1.5) ^§^	Skinfolds	7.2 (1.9)	4.5 (1.1)	Serum	RIA
			101 (58)	134(42)	10.0	10.0	0.62^‡^	0.43^‡^	33 (1.8) ^§^	2.9 (1.6) ^§^	Skinfolds	8.0.8 (2.2)	5.3 (1.4)	Serum	RIA
Nightingale [46]	Cross-sectional	UK	2237(48)	2396(52)	9 to 10^❢^	9;10^❢^	18.6(18.4;18.7)^❡^	18.3(18.1;18.4)^❡^	29.9(29.4;30.4)^❡^	27.2(26.7;27.7)^❡^	Skinfolds	11.5 (11;1)^❡^	7.2 (6.9;7.5)^❡^	Serum	RIA
Thillan [47]	Cross-sectional	Sri Lanka	84 (51)	80 (49)	9.1 (0.3)	9.2 (0.3)	14.9(13.7;16.3)^¶^	14.6 (13.8;17)^¶^	20.7(15.5;27.1)^¶^	16.2(12.6;24.1)^¶^	BIA	3.7(2.1; 6.6)^¶^	2.2 (0.9;5.3)^¶^	Plasma	ELISA

*ELISA* enzyme-linked immunosorbent assay, *RIA* radio immuno assay, *MMA* Milliplex multiplex assays, BMI: body mass index (kg/m2), *DXA* dual-energy X-ray absorptiometry scan; *TOBEC* total body electrical conductivity, *BIA* bioelectrical impedance analysis, *PG* plethysmography, *MPA* SD: standard deviation; weight in kg and SD, BMI z-score^†^; BMI sds^‡^; standard error^§^; median (interquartile range)^¶^; 95% confidence interval^❡^; age range^❢^; median (range)^ℑ^

The body fat percentage was measured by plethysmography (PG) (*n* = 3), bioelectrical impedance (BIA) (*n* = 5), dual-energy X-ray absorptiometry (DXA) (*n* = 5), skinfolds (*n* = 7) and total body electrical conductivity (TOBEC) (*n* = 1). Body index mass (BMI) was reported as kg/m^2^ (*n* = 10), standardised BMI (*n* = 1), BMI Z-score (*n* = 4), and in five studies with newborns and children in the first year of life, weight and height were reported.

### Association of sex with leptin levels

**Fig. 2:**
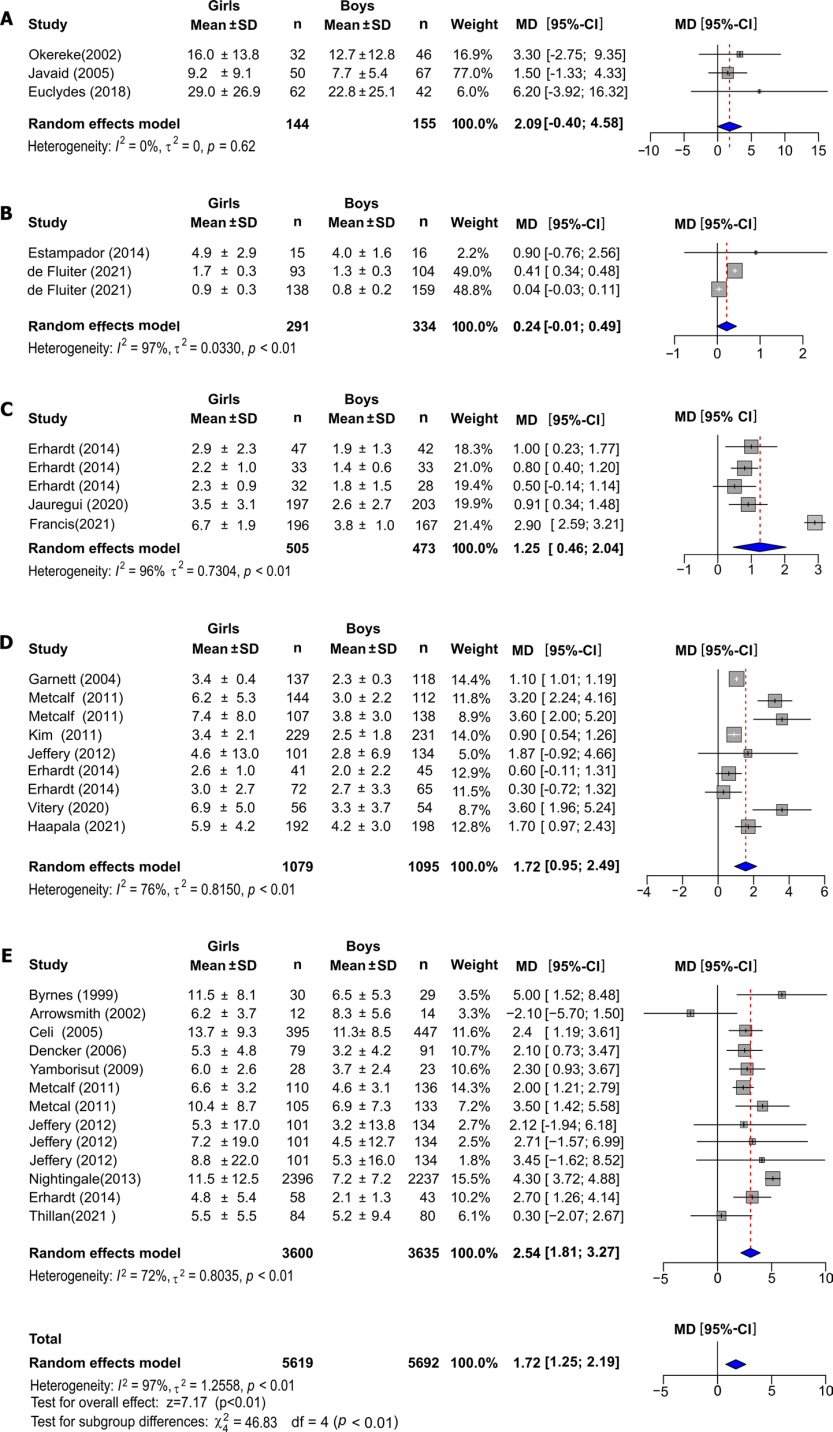
Forest plots of the effect of sex in children's normal-weight on leptin concentrations between 0 and 10 years old. Forest plot showing the overall effect by age groups of newborns (**A**), 0.25–0.5 yrs (**B**), 3–5.9 yrs (**C**), 6–7.9 yrs (**D**), and 8–10 yrs (**E**). Results are presented as mean difference (MD) (95% CI). The study-specific MD and 95% CI are represented by the grey square and horizontal line, respectively. The centre of the blue diamond and the vertical dashed line displays the estimated overall effect size of all studies; the width of the diamond represents the overall pooled 95% CI

The overall effect of sex in the children was associated with higher levels of blood leptin in girls (MD = 1.72 ng/mL, 95% CI: 1.25–2.19, *I*^2^ = 97%), (Fig. 2). The analysis indicated significant differences between age groups (*P* < 0.001). Newborn girls had 2.09 ng/mL higher serum leptin levels than boys, but was not significant (95% CI: − 0.40–4.58, *I*^2^ = 0.0%, *P* = 0.62). Between 0.25 and 0.5 years, boys had 0.24 ng/mL lower leptin (95% CI: − 0.01–0.49 ng/mL, *I*^2^ = 97%, *P* < 0.01). The group 3–5.9 years, the leptin was 1.25 ng/mL (95% CI: 0.46–2.04 ng/mL, *I*^2^ = 96%, *P* = *P* < 0.01) higher in girls, while that the ages of 6–7.9 years and 8–10 years the blood leptin was, respectively, of 1.72 ng/mL (95% CI: 0.95–2.49 ng/mL, *I*^2^ = 76%, *P* < 0.01,) and 2.54 ng/mL (95% CI: 1.81–3.27 ng/mL, *I*^2^ = 72%, *P* < 0.01) higher in girls.

### Association of body fat percentage with sex

**Fig. 3:**
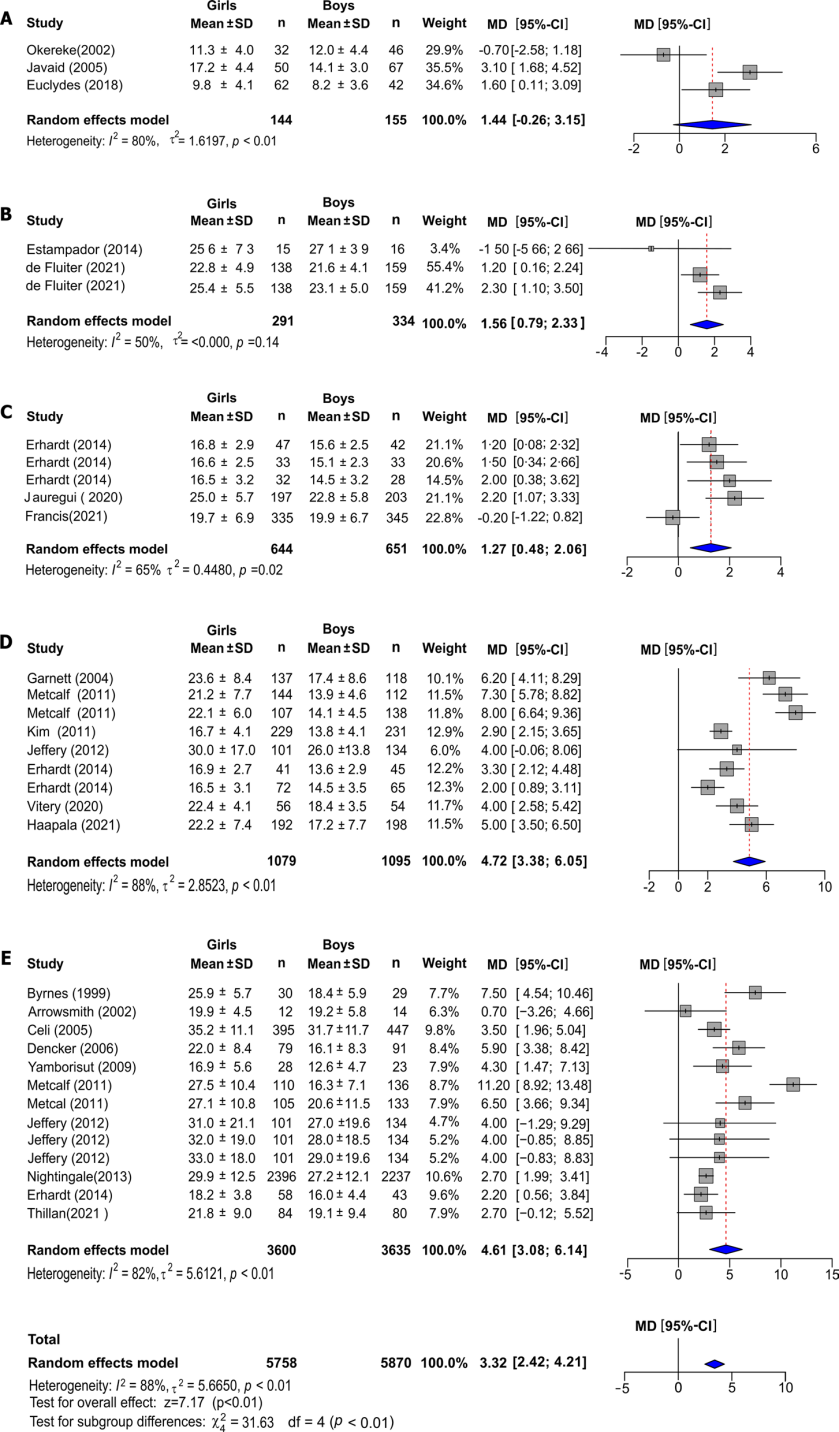
Forest plots of the effect of sex in children's normal-weight on body fat percentage between 0 and 10 years old. Forest plot showing the overall effect by age groups of newborns (**A**), 0.25–0.5 yrs (**B**), 3–5.9 yrs (**C**), 6–7.9 yrs (**D**), and 8–10 yrs (**E**). Results are presented as mean difference (MD) (95% CI). The study-specific MD and 95% CI are represented by the grey square and horizontal line, respectively. The centre of the blue diamond and the vertical dashed line display the estimated overall effect size of all studies; the width of the diamond represents the overall pooled 95% CI

Adiposity was higher in girls in the overall effect (MD = 3.32%, 95% CI: 2.42 to 4.21, *I*^2^ = 88%, P < 0.0001), with significant differences between age groups (*P* < 0.001) (Fig. 3). Meta-analysis showed that body fat percentage was lower in boys except in the newborn group where the association was not significant (MD = 1.44%, 95% CI: − 0.26–3.15, *I*^2^ = 80%, *P* = 0.0976). Boys in the group 0.25–0.5 years had a 1.56% (95% CI: 0.79–2.33%, *I*^2^ = 50%, *P* = 0.14) lower body fat percentage. For the groups of 3–5.9 years, was 1.27% (95% CI: 0.48–2.06%, *I*^2^ = 65%, *P* = 0.02); to 6–7.9 years was 4.72% (95% CI: 3.38–6.05%, *I*^2^ = 88%, *P* < 0.01) and to the group 8–10 years 4.61% (95% CI: 3.08–6.14%, *I*^2^ = 82.4%, *P* < 0.01) higher in girls in all cases.

### Sensitivity analysis

Although there was high heterogeneity in the levels of leptin in the groups of 0.25–0.5 years, 3–5.9 years and 6–7.9 years **(***I*^2^ > 75%), the majority of studies showed blood leptin and body fat percentage higher in girls than in boys across all age groups. In the group of 3–5.9 years, the omitting of results of Francis (2021) [34] showed decreased the heterogeneity and the effect in blood leptin (MD = 0.80 ng/mL, 95% CI: 052 to 1.062 ng/mL, *p* < 0.0001, *I*^2^ = 0%). In the group of 8–10 years, were identified as outliers the studies of Arrowsmith [42] and Nightingale [46], the removal of these studies resulted in an increase of the effect (MD = 2.59 ng/mL, 95% CI: 1.62–3.32 ng/mL, *p* < 0.0001, *I*^2^ = 71.6%). The omitting only of the study of Nightingale [46], resulted in a considerable reduction of heterogeneity (MD = 2.19 ng/mL, 95% CI: 1.72–2.66 ng/mL, *p* < 0.0001, *I*^2^ = 15.8%).

We found a high heterogeneity for body fat percentage in the age groups of newborns, 6–7.9 years, and 8–10 years **(***I*^2^ > 75%). In the newborn group the omitting the study of Okereke [27], resulted in a significant effect of sex on fat (MD = 2.39, 95% CI: 1.35–3.43%, *p* = 0.000, I^2^ = 51.1%). In the children of group 3–5.9 years, the omitting of Francis [34], reduced the heterogeneity in the adiposity (MD = 1.69, 95% CI: 1.08–2.29%, *p* =  < 0.0001, *I*^2^ = 0%). In the group 6–7.9 years old, we identified as outliers the studies of Metcalf [37] and Erhardt [32], the remotion of these two studies increased the effect of sex (MD = 5.20, 95% CI: 3.58 to 6.83, *p* < 0.0001, *I*^2^ = 92.0%). In the group of 8–10 years old, the omitting of one of the groups of Metcalf [37], resulted in a reduction of heterogeneity (MD = 3.84, 95% CI: 2.81–4.87%, *p* < 0.0001, *I*^2^ = 53.6%) (Additional file 1: Tables S1 and S2).

Sensibility analysis indicated that the overall statistical significance did not change when any single study or several outliers were omitted. Therefore, the results of this meta-analysis are deemed to be relatively reliable and credible.

### Subgroup analyses of plasma leptin levels and body fat percentage

To examine the influence of certain characteristics of the studies selected on blood leptin and body fat percentage, we carried out a subgroup analysis on the overall effect. Considering the study design, we found that the cross-sectional studies (MD = 1.94, 95% CI = 1.05–2.82, *I*^2^ = 90.8%, p < 0.01) showed a higher difference by sex than the cohort studies (MD = 1.58, 95% CI = 1.05–2.82 ng/mL, *I*^2^ = 90.8%, *p* < 0.01). The results indicated both for the measurements of leptin by ELISA (MD = 0.62 ng/mL, 95% CI = 0.39–1.09 ng/mL, *I*^2^ = 58.0%, *p* < 0.01) and RIA (MD = 2.23 ng/mL, 95% CI = 1.64–2.82 ng/mL, *I*^2^ = 88.9.1%, *p* < 0.01) the girls had higher leptin. Lower ELISA values can be attributed to the fact that most of the studies that used this technique were in children under 6 years of age. The subgroup analysis by each region also showed higher leptin blood in girls in each group (Additional file 1: Table S3). Studies with high quality (*n* = 17) showed that girls had higher leptin (MD = 1.86 ng/mL, 95% CI = 1.37–2.34 ng/mL, *I*^2^ = 86.7%, *p* < 0.01) while the studies with low quality (*n* = 4) did not show this effect (MD = 0.78 ng/mL, 95% CI = − 0.21–1.77 ng/mL, *I*^2^ = 94.7%, *p* < 0.01).

According to the design study, body fat percentage was higher in girls, with a larger effect in studies type cohort (MD = 3.86%, 95% CI = 2.64–5.08%, *I*^2^ = 90.5%, *p* < 0.01) than cross-sectional (MD = 2.79%, 95% CI = 1.85 to 3.72, *I*^2^ = 68.1%, *p* < 0.01). Independent of the method of measurement and geographic region, boys had lower body fat percentage, while quality assessments indicated a similar effect for adiposity in the studies with low and high quality (Additional file 1: Table S4).

### Publication bias

The funnel plots were symmetrical and Eager tests were not significant (*P* > 0.05), for blood leptin and body fat percentage in the groups of 6–7.9 year and 8–10 years that included ≥ 6 reports (Additional file 1: Figs. S1 and S2).

## Discussion

This systematic review/meta-analysis to our knowledge is the first to describe in normal-weight children population an association of sex with blood leptin and body fat percentage before ten years old. Our analysis shows these adiposity indicators are higher in girls and that both sexes increase progressively during the first 10 years, with an apparent rate higher in the girls.

Currently, the mechanisms by which the prepubescent girls with normal-weight have higher leptin and fat than the boys remain underestimated, however, the results of some studies suggest that the early difference in sexual hormone could be implicated. The study of Garcia-Mayor et al. in Spanish children (5–15 years old) found a continuous increase in the levels of leptin in prepubertal girls of 6–10 years of age, parallel with changes in levels of FSH, without changes in the estradiol and LH hormones, however, this study did not measure the fat body in the subjects [48]. Blum et al. reported only in boys an inverse correlation between leptin with testosterone, this hormone explained about the 10% of the variation of leptin in males after adjusting by age and IBM [49]; however, this study included in the analysis population in period pubertal that can influence this association. Studies in vitro in cell culture of adipocytes and explants of adipose tissue have shown that androgen-like dihydrotestosterone, represses the transcription of the leptin gene, while low doses of estradiol stimulate abundant expression [49–51]. Therefore, it is possible that androgens in boys can reduce the level of leptin, while in girls the estrogens could increase their production.

The previous reports indicate that androgen and estrogens could be determinants to maintain the sexual dimorphism of leptin, at least during puberty and adulthood. Studies on girls and boys before 10 years old have already reported differences in levels of some androgens and estrogens. Courant et al. reported in children between 6 and 8 years old, levels of 17β-estradiol were higher in girls, while that prepubertal boys of the same age had higher levels of 17α-testosterone [52]. Frederiksen et al. found that girls between 0 and 4 years had higher levels of estrone and estradiol than boys [53]. Therefore, differences in sex steroid hormones during prepuberty could be associated with the leptin behaviour observed in both sexes in our study, although the levels of these hormones could be too low in children to explain these differences.

Among the mechanisms that could be related to higher circulating leptin in girls are epigenetic modifications that can affect gene expression. Dunstan et al. found in children of 10–15 years old, differences in leptin promoter DNA methylation (*LEP*) by sex in four sites CpG [54]. Lesseur et al. reported a significant association between sex and *LEP* methylation in the placenta, with higher methylation in males compared to females [55]. Additionally, methylation at the *LEP* promoter has shown an inverse relationship with leptin tissue expression in human cells in vitro and primary tissue [56, 57]; therefore, the contribution of this type of modification could be considered to explain the sex differences in blood leptin in children.

In adults, leptin levels have been related with sex-specific fat distribution, particularly the subcutaneous adipose tissue has been strongly associated with blood leptin and weakly with visceral fat [58, 59]. Some studies have shown that girls have more peripheral fat and less visceral fat than boys [60, 61]. The pattern of fat accumulation in children could be related to the capacity of synthesis of leptin by the adipocytes. Nagy et al*.* found in children between 6 and 10 years of age that by adjusting blood leptin by body fat distribution, the leptin was no longer associated with sex, suggesting that the sexual dimorphism of this adipokine may be due to the composition body relative and the distribution of fat [62].

Leptin has been implicated in the regulation of several functions which has made the interpretation of its metabolic and physiological interactions difficult [63]. However, it has been proposed that the chronic elevation of blood leptin may be associated with altered signalling of both insulin and leptin [64, 65], dysregulation of lipid metabolism [66], blood pressure, and kidney diseases [67, 68]. Leptin exerts dichotomous and paradoxical effects on cardiovascular function, in most cases, high leptin levels correlate positively with unfavourable outcomes [69, 70], such as decreased arterial distensibility or atherosclerosis, which has been associated with macrovascular diseases, and increased risk of myocardial infarction [71]. The early dimorphism in which leptin levels are higher in girls may favour the development of alterations in its signalling and the effects [72].

It has been suggested that cardiometabolic risk in adolescence can be predicted from the trajectory of leptin during childhood [82], a trajectory with intermediate values at birth followed by an increase in mid-childhood has been associated with several risk markers [83]. The increased leptin level in mid-childhood could correspond to the fat rebound that occurs at these ages; however, the causes of this rebound are still unknown. Our results showed a greater difference in leptin levels by sex in the adipose rebound period (4 to 7 years old), which also coincides with an increase in body fat percentage [84], which is consistent with some reports indicating that girls have adipose rebound at younger ages than boys [85] and that this, in turn, is associated with a higher metabolic risk [84, 86].

Our results of body fat percentage are similar to other studies not included in this meta-analysis (did not meet the inclusion criteria) and that reported significantly higher fat mass in prepubertal girls [73–76]. Among the explanations that may support higher fat in girls is higher energy intake at younger ages (18 months) associated at older ages (9 years) with a higher fat mass in girls, while in boys with an increase in the free fat mass [77, 78]. Another finding that could be related to higher adiposity in girls, is a lower physical activity and a higher sedentary behaviour compared to boys of similar age. It has been documented in several populations around the world: Asia [79–81], Europe [82, 83], the United States [84], and Australia [85]. These studies indicate that the differences in physical activity between boys and girls are observed at all ages, which is less marked as they are younger. The expenditure of energy associated with physical activity and the higher energy intake could contribute to explaining the differences in fat mass. Then, fat and leptin dimorphism between girls and boys, could arise as consequence of physical activity level or sedentary time. It should be mentioned that among the articles that were selected, only three studies reported physical activity levels, though without specifying by sex [33, 40, 47].

Although usually sex differences in adiposity in adulthood have been related with gonadal hormones, murine models had shown that sex chromosome complement (XX vs XY) influences body weight and adiposity independent of these hormones [86], which has been attributed to the effect of the X chromosome dosage, particularly to the subset of X chromosome genes that escape to epigenetic inactivation and that exhibit higher expression levels in adipose tissue [87]. Recently Link et al. reported an association sex-dependent dosage of gen Kdm5ct expressed in chromosome X related to higher adipogenesis in females [88]. However, it is still unknown whether these biological mechanisms contribute to early sexual dimorphism of adiposity in humans.

We found high heterogeneity of the effect of sex on leptin level and body fat percentage in some age groups, which can be related to biological factors. For instance, at birth leptin appears to be strongly influenced by maternal weight and BMI before pregnancy, as well as by other maternal variables during gestation such as diabetes, smoking, and level of physical activity [89, 90]. Leptin levels in early childhood also appear to be affected by nutritional behaviour and physical activity [91], as was mentioned above. Ethnic variations in circulating leptin and body fat percentage also have been reported in children and adulthood, even after being adjusted by IBM [92–95]. However, our analysis showed consistent results during the first 10 years of life, with higher leptin and fat in girls.

The present systematic review/meta-analysis has several strengths. Our study covered a comprehensive range of ages from birth to ten years, such as the period of rebound adipose (4–7 years old), avoiding the influence of the hormonal changes that occur at puberty. Importantly, we focused on studies with populations of normal-weight children, without the influence of conditions such as obesity or underweight. Besides, the analysis of sensibility did not show that the differences by sex were modified by factors such as the design of the study, the technique of measurement, geographical area and outlier reports. Some limitations of this research deserve to be mentioned: a high heterogeneity found among the reports selected, could be explained by the methodological characteristics and biological factors of the populations as was discussed above. For some age groups such as newborns, 0.25 to 5 years and 3 to 5.9 years, we found few studies with small size samples, which may affect the estimation of the effect of these groups. Should also be mentioned that we did not find studies from certain regions such as Africa.

## Perspectives and significance

This systematic review/meta-analysis contravenes the idea that differences in blood leptin concentration and body fat percentage begin during puberty; on the contrary, our results support that the girls present higher values of these adiposity markers than boys from prepuberty. The biological significance, mechanisms, and physiological consequences on human health of this early sexual dimorphism should be studied considering that paediatric populations are vulnerable to metabolic alterations, underweight, overweight and obesity.

The prepubertal period is a critical stage for the development of fat tissue, since it is during this period that adipose rebound occurs, a process whose early onset in children has been linked to the development of components of metabolic syndrome in childhood, adolescence, and adulthood [96–98]. Among the factors that may be related to the onset of adipose rebound are the trajectories of fat tissue and blood leptin before the rebound, these factors have not been sufficiently studied before this stage, although these trajectories in older children have been associated with the development of metabolic diseases at later stages, and these associations have been stronger when adjusted for sex [99–101]. This suggests that sexual dimorphism of fat tissue and leptin should be considered from an early age for the design and analysis of future research involving prepubertal children, which will contribute to improving knowledge, understanding and prevention of metabolic diseases from childhood.

## Supplementary Information

**Additional file 1.** Additional tables and figures.
