# Correction: Sexual dimorphism of leptin and adiposity in children between 0 and 10 years: a systematic review and meta-analysis

**DOI:** 10.1186/s13293-022-00478-4

**Published:** 2022-11-10

**Authors:** Jose Guillermo Ortega-Avila, Harry García-Muñoz, Alejandro Segura Ordoñez, Blanca C. Salazar Contreras

**Affiliations:** 1 Grupo de Investigación de Ciencias Básicas y Clínicas de la Salud, Departamento de Ciencias Básicas de la Salud, Pontifcia Universidad Javeriana, Seccional-Cali, Cali, Colombia; 2 Facultad de Salud, Grupo de Investigación Salud y Movimiento, Universidad Santiago de Cali, Cali, Colombia; 3 Grupo de Nutrición, Departamento de Ciencias Fisiológicas, Facultad de Salud, Universidad del Valle, Cali, Colombia; 4 Programa de Medicina, Facultad de Salud, Universidad Icesi, Cali, Colombia

## Correction: Biology of Sex Differences (2022) 13:47 https://doi.org/10.1186/s13293-022-00454-y

Following publication of the original article [1], the authors reported an error in the affiliations.

Harry García-Muñoz is affiliated to 2, 3, 4.

Blanca C. Salazar Contreras is affiliated to 3.

The author group above has been corrected to reflect the updated affiliations. The original article [1] has been corrected.
