# Supplementary material for: Sexual dimorphism of leptin and adiposity in children between 0 and 10 years: a systematic review and meta-analysis
Source: Biol Sex Differ. 2022 Sep 5;13:47. doi: 10.1186/s13293-022-00454-y (PMC9446796; doi:10.1186/s13293-022-00454-y)
Supplement: Supplementary file 1 — Additional file 1. Additional tables and figures. [file 13293_2022_454_MOESM1_ESM.docx]

Supplemental

**Algorithm Search by database**

OVID (Medline):

(exp leptin/ or leptin.mp. or (obese adj2 protein).mp.) and (exp adiposity/ or adiposity.mp. or obesity.mp. or exp body composition/ or body composition.mp. or exp Obesity, Abdominal/ or obesity abdominal.mp. or exp Pediatric Obesity/ or pediatric obesity.mp. or body adiposity index.mp. or bmi trajectory.mp. or exp Body Mass Index/ or body mass index.mp. or exp Waist Circumference/ or waist circumference.mp. or fat mass.mp. or visceral adiposity index.mp. or exp Intra-Abdominal Fat/ or visceral fat.mp. or fat thickness.mp. or body fat percentage.mp. or anthropometric indices.mp. or body shape index.mp. or anthropometric parameter*.mp. or obesity indices.mp. or triceps skinfold thickness.mp.) and (exp Infant/ or infant.mp. or exp child/ or child.mp. or children.mp. or exp adolescent/ or adolescent.mp)

EMBASE:

('leptin'/exp OR leptin) AND ('adiposity'/exp OR adiposity OR 'obesity'/exp OR obesity OR 'body composition'/exp OR 'body composition' OR 'abdominal obesity'/exp OR 'abdominal obesity' OR 'pediatric obesity'/exp OR 'pediatric obesity' OR 'body adiposity index'/exp OR 'body adiposity index' OR 'bmi trajectory' OR 'body mass index'/exp OR 'body mass index' OR 'waist circumference'/exp OR 'waist circumference' OR 'fat mass'/exp OR 'fat mass' OR 'visceral adiposity index'/exp OR 'visceral adiposity index' OR 'visceral fat'/exp OR 'visceral fat' OR 'fat thickness'/exp OR 'fat thickness' OR 'body fat percentage'/exp OR 'body fat percentage' OR 'anthropometric indices' OR 'body shape index'/exp OR 'body shape index' OR 'anthropometric parameter*' OR 'obesity indices' OR 'triceps skinfold thickness'/exp OR 'triceps skinfold thickness') AND ('infant'/exp OR infant OR 'child'/exp OR child OR 'children'/exp OR children OR 'adolescent'/exp OR adolescent)

LILACS:

(tw:(LEPTINA)) AND (tw:(adiposidad OR obesidad OR "composición corporal" OR "obesidad abdominal" OR "obesidad pediatrica" OR "índice de adiposidad corporal" OR "trayectoria de IMC" OR "indice de masa corporal" OR "circunferencia de cintura" OR "masa grasa" OR "adiposidad visceral" OR "grasa visceral" OR "porcentaje de grasa corporal" OR "índices antropométricos" OR "parámetros antropométricos" OR "índice de obesidad" OR "pliegue cutáneo de tríceps")) AND (tw:(infancia OR niñez OR niños OR adolescentes))

**Supplemental Figure S1.** Funnel plots of leptin by age groups **A** 6-7.9 yrs, **B.** 8–10 yrs.

1. **B.**
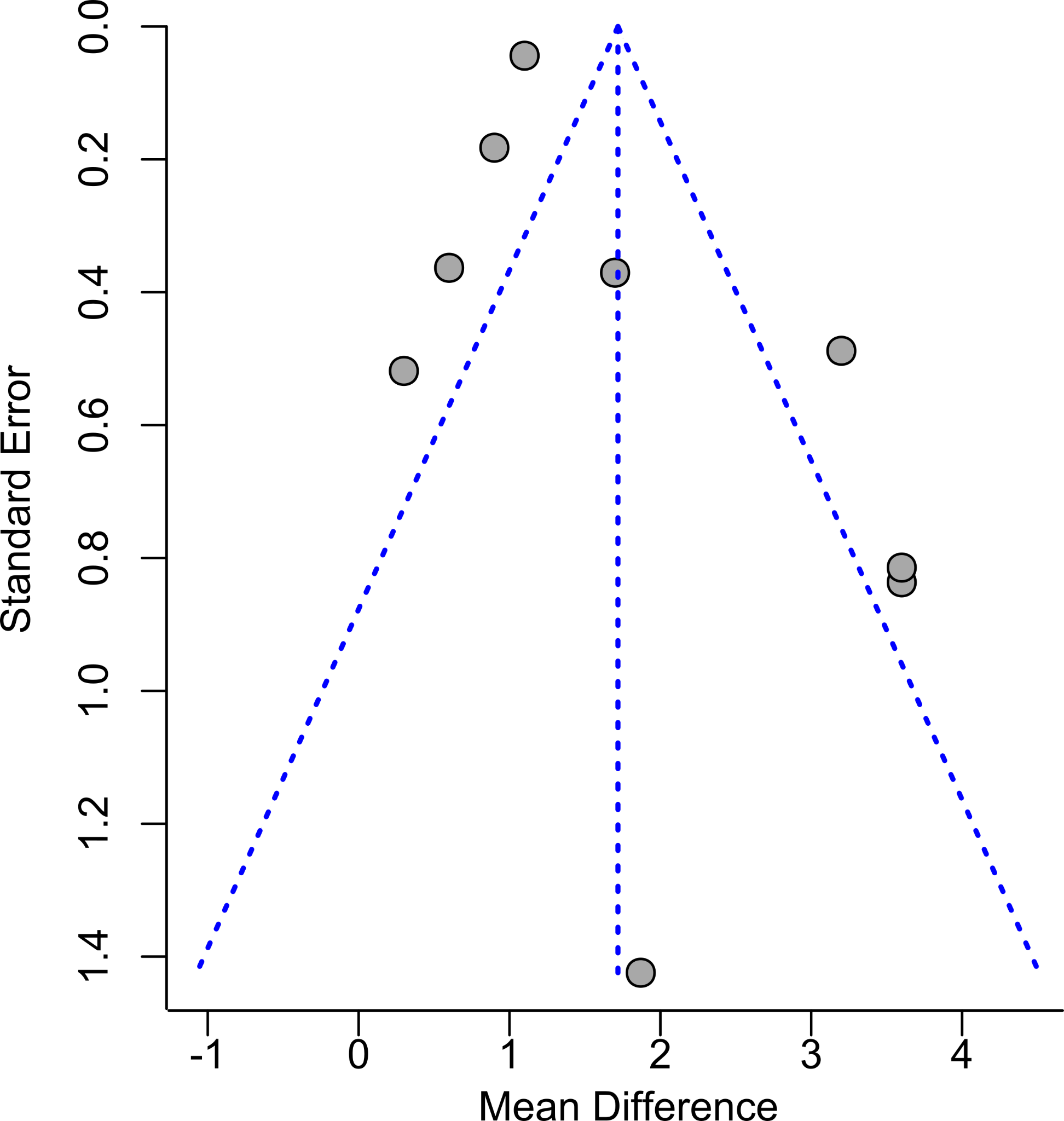

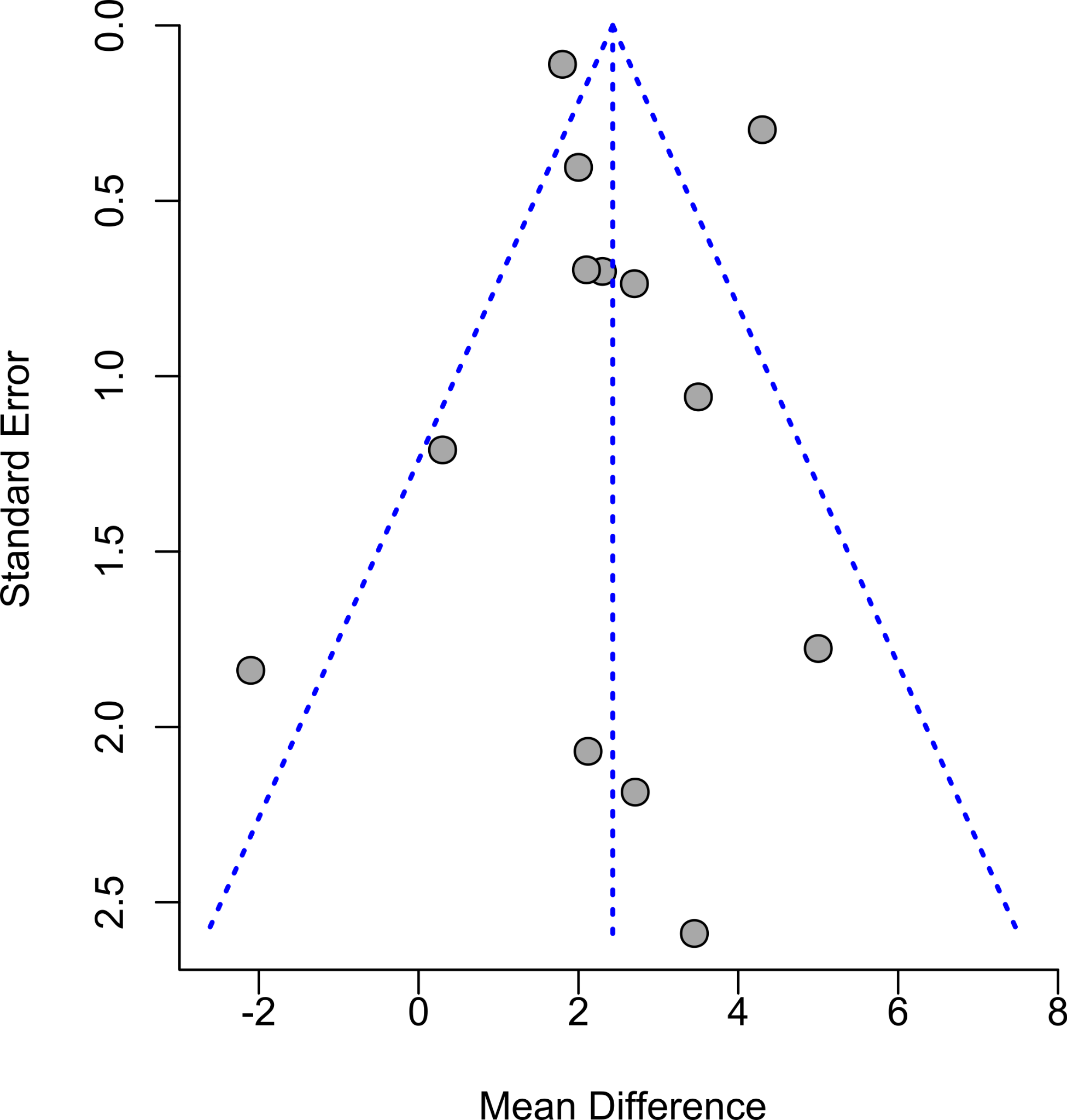


**B.**

**Supplemental Figure S2.** Funnel plots body fat percentage by age groups, **A.** 6-7.9 yrs, **B.** 8–10 yrs.

1. **B.**
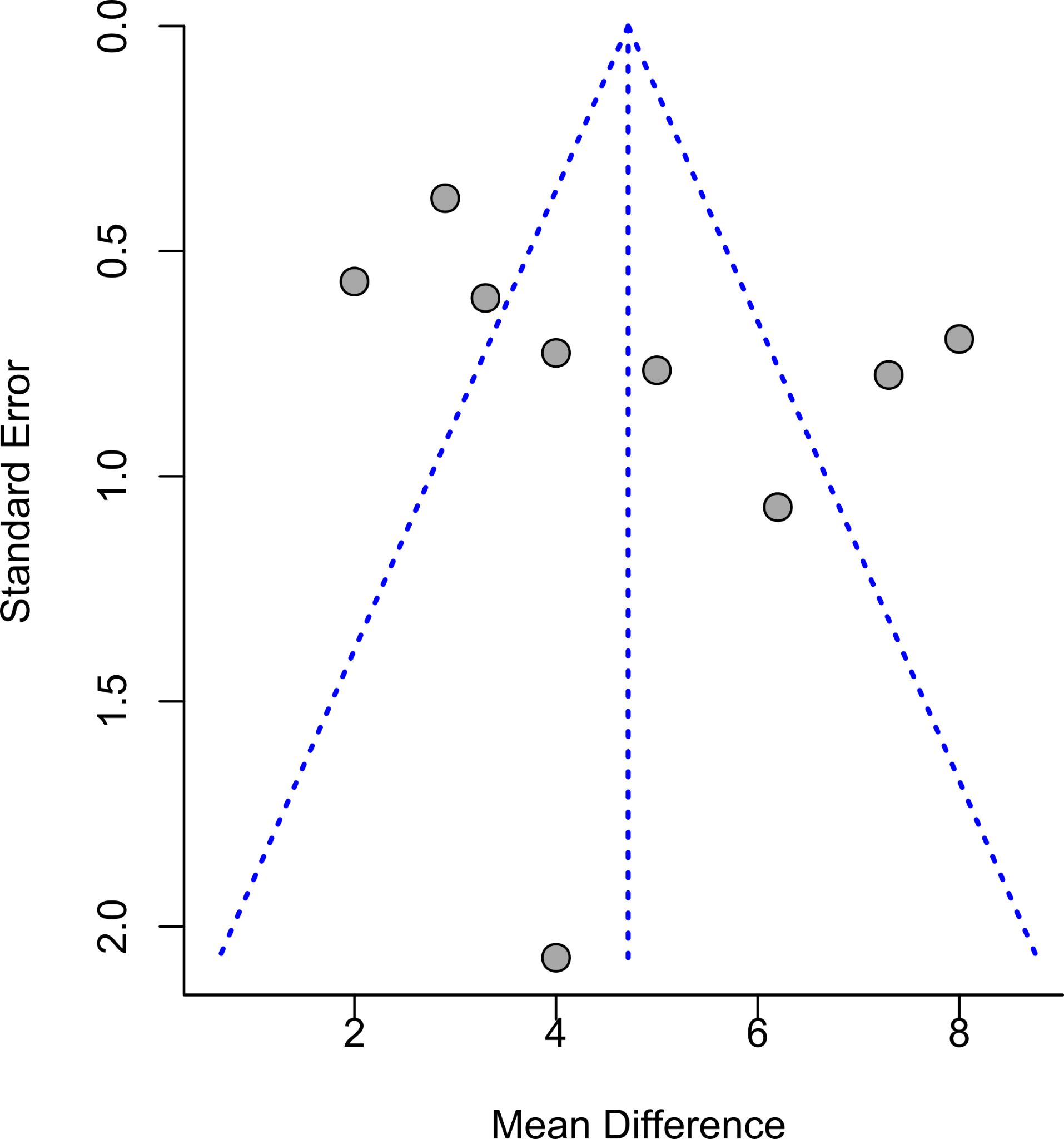

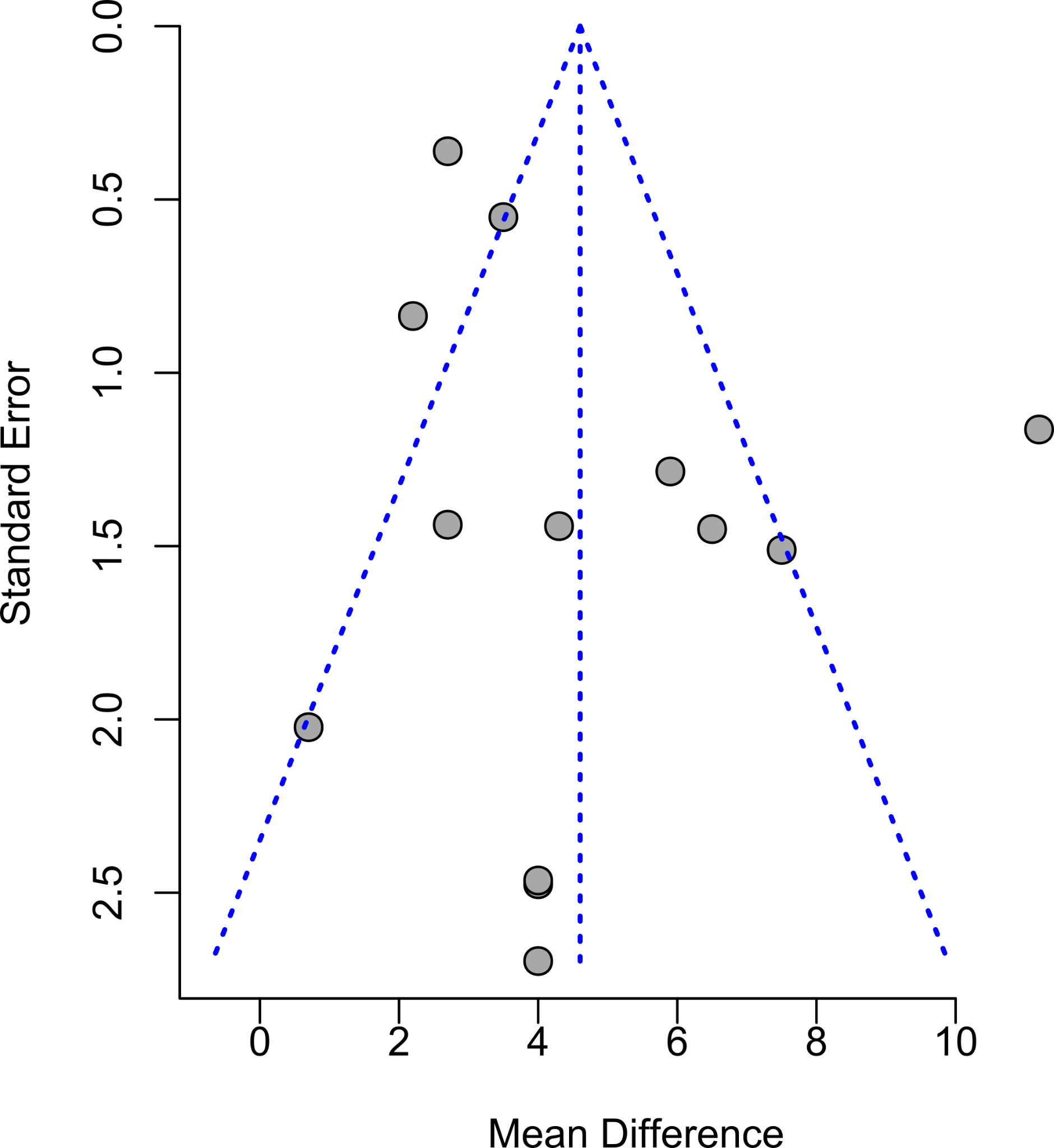


**Supplemental Table S1.** Leave-one-out sensitivity analysis for blood leptin by subgroup age.

| **Age group** | **Study omitted** | **MD** | **IC-95%** | **P value** | **I^2^%** |
| --- | --- | --- | --- | --- | --- |
| **Newborns** | Okereke (2002) | 1.84 | -0.89;4.57 | 0.1860 | 0.0 |
|  | Javaid (2005) | 4.06 | -1.13;9.25 | 0.1250 | 0.0 |
|  | Euclydes (2018) | 1.82 | -0.74;4.39 | 0.1635 | 0.0 |
|  | Pooled estimate | 2.09 | -0.40; 4.58 | 0.0998 | 0.0 |
| **0.25 to 0.5 years** | Estampador (2014) | 0.22 | -0.03;0.48 | 0.0851 | 98.3 |
|  | de Fluiter (2021) | 0.04 | -0.02;0.11 | 0.2285 | 2.4 |
|  | de Fluiter (2021) | 0.41 | 0.35;0.48 | < 0.0001 | 0.0 |
|  | Pooled estimate | 0.24 | -0.01;0.49 | 0.063 | 96.7 |
| **3 to 5.9 years** | Erhardt (2014) | 1.30 | 0.35;2.25 | 0.0073 | 97 |
|  | Erhardt (2014) | 1.37 | 0.41;2.32 | 0.0049 | 96 |
|  | Erhardt (2014) | 1.43 | 0.54;2.32 | 0.0016 | 96.6 |
|  | Jauregui (2020) | 1.33 | 0.37;2.29 | 0.0065 | 96.8 |
|  | Francis (2021) | 0.79 | 0.52; 1.06 | < 0.0001 | 0 |
|  | Pooled estimate | 1.25 | 0.46;2.04 | 0.0019 | 97.0 |
| **6 to 7.9 years** | Haapala (2021) | 1.74 | 0.85;2.64 | 0.0001 | 83.6 |
|  | Vitery (2020) | 1.51 | 0.79;2.25 | < 0.0001 | 80.7 |
|  | Metcalf (2011) | 1.47 | 0.76;2.18 | < 0.0001 | 73.8 |
|  | Metcalf (2011) | 1.51 | 0.78;2.23 | < 0.0001 | 80.4 |
|  | Kim (2011) | 1.86 | 0.99;2.72 | < 0.0001 | 84.0 |
|  | Jeffery (2012) | 1.71 | 0.90;2.53 | < 0.0001 | 84.4 |
|  | Garnett (2004) | 1.83 | 0.95;2.72 | < 0.0001 | 84.1 |
|  | Erhardt (2014) | 1.88 | 1.05;2.71 | < 0.0001 | 83.8 |
|  | Erhardt (2014) | 1.89 | 1.09;2.69 | < 0.0001 | 83.6 |
|  | Pooled estimate | 1.72 | 0.95;2.49 | < 0.0001 | 76.0 |
| **8-10 years** | Yamborisut (2009) | 2.54 | 1.62;3.24 | < 0.0001 | 74.5 |
|  | Metcalf (2011) | 2.60 | 1.79;3.42 | < 0.0001 | 68.8 |
|  | Metcal (2011) | 2.45 | 1.67;3.22 | < 0.0001 | 74.5 |
|  | Jeffery (2012) | 2.54 | 1.79;3.29 | < 0.0001 | 74.5 |
|  | Jeffery (2012) | 2.52 | 1.78;3.25 | < 0.0001 | 74.6 |
|  | Jeffery (2012) | 2.51 | 1.77;3.25 | < 0.0001 | 74.6 |
|  | Erhardt (2014) | 2.49 | 1.68;3.30 | < 0.000 | 74.5 |
|  | Dencker (2006) | 2.57 | 1.77;3.37 | < 0.0001 | 73.5 |
|  | Celi (2005) | 2.53 | 1.71;3.35 | < 0.0001 | 74.0 |
|  | Byrnes (1999) | 2.44 | 1.70;3.19 | < 0.0001 | 73.9 |
|  | Arrowsmith (2002) | 2.70 | 2.01;3.39 | < 0.0001 | 69.0 |
|  | Thillian (2021) | 2.69 | 1.98;3.40 | < 0.0001 | 71.2 |
|  | Nightingale (2013) | 2.19 | 1.72;2.66 | < 0.0001 | 15.8 |
|  | Pooled estimate | 2.54 | 1.81;3.27 | < 0.000 | 72.0 |

**Supplemental Table S2.** Leave-one-out sensitivity analysis for body fat percentage by subgroup age.

| **Age group** | **Study omitted** | **MD** | **IC-95%** | **p-value** | **I^2^%** |
| --- | --- | --- | --- | --- | --- |
| **Newborns** | Okereke (2002) | 2.39 | 1.35;3.43 | 0.000 | 51.1 |
|  | Javaid (2005) | 0.6 | -0.97;2.17 | 0.452 | 71.6 |
|  | Euclydes (2018) | 1.31 | -1.32;3.93 | 0.330 | 90.0 |
|  | Pooled estimate | 1.44 | -0.26;3.15 | 0.098 | 80.0 |
| **0.25 to 0.5 years** | Estampador (2014) | 1.67 | 0.88;2.45 | < 0.0001 | 45.8 |
|  | de Fluiter (2021) | 2.00 | 0.85; 3.16 | 0.0007 | 66.2 |
|  | de Fluiter (2021) | 1.04 | 0.03;2.05 | 0.0423 | 34.4 |
|  | Pooled estimate | 1.56 | 0.79; 2.33 | < 0.0001 | 50.0 |
| **3 to 5.9 years** | Erhardt (2014) | 1.30 | 0.31;2.29 | 0.0101 | 73.7 |
|  | Erhardt (2014) | 1.22 | 0.25;2.20 | 0.0141 | 72.9 |
|  | Erhardt (2014) | 1.14 | 0.26;2.02 | 0.0108 | 71 |
|  | Jauregui (2020) | 1.00 | 0.18;1.83 | 0.0166 | 60.1 |
|  | Francis (2021) | 1.69 | 1.08; 2.29 | < 0.0001 | 0 |
|  | Pooled estimate | 1.26 | 0.48;2.06 | 0.0017 | 65% |
| **6 to 7.9 years** | Haapala (2021) | 4.68 | 3.17;6.18 | < 0.0001 | 91.0 |
|  | Vitery (2020) | 4.81 | 3.31;6.31 | < 0.0001 | 91.1 |
|  | Metcalf (2011) | 4.37 | 3.03;5.7 | < 0.0001 | 88.4 |
|  | Metcalf (2011) | 4.24 | 3.03;5.45 | < 0.0001 | 84.1 |
|  | Kim (2011) | 4.98 | 3.54;6.41 | < 0.0001 | 89.2 |
|  | Jeffery (2012) | 4.76 | 3.34;6.19 | < 0.0001 | 91.1 |
|  | Garnett (2004) | 4.54 | 3.1;5.99 | < 0.0001 | 90.7 |
|  | Erhardt (2014) | 4.91 | 3.44;6.38 | < 0.0001 | 90.9 |
|  | Erhardt (2014) | 5.09 | 3.77;6.41 | < 0.0001 | 88.9 |
|  | Pooled estimate | 4.72 | 3.38;6.05 | < 0.0001 | 88.0 |
| **8-10 years** | Yamborisu (2009) | 4.63 | 2.98;6.27 | < 0.0001 | 83.8 |
|  | Metcalf (2011) | 3.84 | 2.81;4.87 | < 0.0001 | 53.6 |
|  | Metcal (2011) | 4.44 | 2.82;6.07 | < 0.0001 | 82.9 |
|  | Jeffery (2012) | 4.63 | 3.03;6.24 | < 0.0001 | 83.9 |
|  | Jeffery (2012) | 4.64 | 3.03;6.25 | < 0.0001 | 83.9 |
|  | Jeffery (2012) | 4.64 | 3.03;6.25 | < 0.0001 | 83.9 |
|  | Erhardt (2014) | 4.86 | 3.26;6.47 | < 0.0001 | 83.1 |
|  | Dencker (2006) | 4.49 | 2.84;6.13 | < 0.0001 | 83.1 |
|  | Celi (2005) | 4.72 | 3.05;6.39 | < 0.0001 | 83.9 |
|  | Byrnes (1999) | 4.36 | 2.78;5.95 | < 0.0001 | 82.1 |
|  | Arrowsmith (2002) | 4.86 | 3.31;6.41 | < 0.0001 | 83.4 |
|  | Thillan(2021 ) | 4.77 | 3.15;6.39 | < 0.0001 | 83.8 |
|  | Nightingale (2013) | 4.83 | 3.19;6.47 | < 0.0001 | 79.8 |
|  | Pooled estimate | 4.61 | 3.08;6.14 | < 0.0001 | 82.0 |

**Supplemental Table S3.** Subgroup analyses for the overall effect of sex on blood leptin concentrations according to study characteristics.

|  |  | **No. Studies/ No. Age group** | **MD (95% CI)** | **P-value** | **P heterogeneity** | **I^2^%** | **P for difference between subgroups** |
| --- | --- | --- | --- | --- | --- | --- | --- |
| **Design** | Cohort | 10/22 | 1.58 (1.05; 2.10) | < 0.0001 | < 0.01 | 97.0 | 0.4906 |
|  | Cross-sectional | 11/11 | 1.94 (1.05; 2.82) | < 0.0001 | < 0.01 | 90.8 |  |
| **Method** | RIA | 14/20 | 2.23(1.64; 2.82) | < 0.0001 | < 0.01 | 88.9 | 0.0001 |
|  | ELISA | 5/10 | 0.62(0.39; 1.09) | < 0.0001 | < 0.01 | 58.0 |  |
|  | MMA | 2/3 | 1.10(-0.32; 2.53) | 0.1289 | < 0.01 | 99.6 |  |
| **Region** | America | 5/5 | 2.4 (2.2; 3.64) | 0.0361 | < 0.01 | 89.7 | 0.0260 |
|  | Asia | 3/3 | 0.97 (0.63; 1.31) | < 0.0001 | 0.13 | 50.6 |  |
|  | Europe | 10/22 | 1.70 (1.13; 2.27) | < 0.0001 | < 0.01 | 94.6 |  |
|  | Oceania | 3/3 | 0.97 (0.63;1.32) | < 0.0001 | 0.0198 | 74.5 |  |
| **Quality** | High | 17/27 | 1.86 (1.37; 2.34) | < 0.0001 | < 0.01 | 86.7 | < 0.0001 |
|  | Low | 4/5 | 0.78 (-0.21; 1.77) | 0.1254 | < 0.01 | 94.7 |  |
| **Overall** |  | 21/33 | 1.72(1.25; 2.19) | < 0.0001 | < 0.01 | 97.0 | - |

**Supplemental Table S4.** Subgroup analyses for the overall effect of sex on body fat percentage according to study characteristics.

|  |  | **No. Studies/ Age group** | **MD (95% CI)** | **P-value** | **P heterogeneity** | **I^2^%** | **P for difference between subgroups** |
| --- | --- | --- | --- | --- | --- | --- | --- |
| **Design** | Cohort | 10/22 | 3.86(2.64; 5.08) | < 0.0001 | < 0.01 | 91.2 | 0.1719 |
|  | Cross-sectional | 11/11 | 2.79(1.85; 3.72) | < 0.0002 | < 0.01 | 68.1 |  |
| **Method** | BIA | 4/5 | 3.4 (2.14; 4.73) | < 0.0001 | < 0.01 | 65.8 | < 0.0001 |
|  | DXA | 6/8 | 6.58 (5.04; 8.13) | < 0.0001 | < 0.01 | 85.4 |  |
|  | Plethysmography | 4/5 | 1.15 (0.30; 2.0) | 0.0078 | 0.0628 | 55.2 |  |
|  | Skinfolds | 6/14 | 2.49 (1.96; 3.03) | < 0.0001 | 0.07 | 33.6 |  |
| **Region** | America | 5/5 | 1.44 (-0.01; 2.90) | 0.0521 | < 0.01 | 83.4 | 0.0373 |
|  | Asia | 3/3 | 2.97 (2.27; 3.67) | < 0.0001 | 0.63 | 0.0 |  |
|  | Europe | 11/22 | 3.77(2.64; 4.90) | < 0.0001 | < 0.0001 | 89 |  |
|  | Oceania | 3/3 | 5.20 (2.35; 8.05) | < 0.0001 | 0.02 | 74.2 |  |
| **Quality** | High | 17/28 | 3.42 (2.44; 4.40) | < 0.0001 | < 0.01 | 88.9 | 0.9994 |
|  | Low | 4/5 | 3.42 (1.21; 5.64) | 0.0024 | < 0.01 | 83.8 |  |
| **Overall** |  | 21/33 | 3.32 (2.42; 4.21) | < 0.0001 | < 0.0001 | 88.0 | - |

**Supplemental Table S5**. Quality of included studies in the systematic review/ meta-analysis.

|  | **Selection** | | | | **Comparability** | **Outcome** | | **Total** |
| --- | --- | --- | --- | --- | --- | --- | --- | --- |
|  | Maximal 5 ★ | | | | Maximal 2 ★ | Maximal 3 ★ | | Maximal 10 ★ |
| **Author (Year)** | **REP** | **SAM** | **NREP** | **AE** | **COM** | **ASS** | **STAT** |  |
| Byrnes (1999) | **★** | **-** | **-** | **★★** | **-** | **★★** | **★** | 6 |
| Arrowsmith (2002) | **★** | **-** | **★** | **★** | **-** | **★** | **★** | 5 |
| Ndubueze (2002) | **★** | **★** | **★** | **★★** | **★** | **★★** | **★** | 9 |
| Garnett (2004) | **★** | **★** | **-** | **★★** | **★** | **★★** | **★** | 8 |
| Celi(2005) | **★** | **★** | **★** | **★★** |  | **★★** | **★** | 8 |
| Javaid (2005) | **★** | **★** | **★** | **★★** | **★** | **★★** | **★** | 9 |
| Dencker(2006) | **★** | **-** | **-** | **★★** | **-** | **★★** | **★** | 6 |
| Yamborisut (2009) | **★** | **★** | **★** | **★★** | **★** | **★★** | **★** | 9 |
| Kim (2011) | **★** | **★** | **★** | **★** | **★** | **★★** | **★** | 9 |
| Metcalf (2011) | **★** | **★** | **-** | **★★** | **-** | **★★** | **★** | 7 |
| Jeffery (2012) | **★** | **★** | **★** | **★★** | **★** | **★★** | **★** | 9 |
| Nightingale (2013) | **★** | **★** | **-** | **★★** | **-** | **★★** | **★** | 7 |
| Erhardt (2014) | **★** | **-** | **★** | **★** | **★★** | **★★** | **★** | 8 |
| Estampador (2014) | **★** | **★** | **★** | **★★** | **★** | **★★** | **★** | 9 |
| Euclydes (2018) | **★** | **★** | **★** | **★★** | **★** | **★★** | **★** | 9 |
| Jauregui (2020) | **★** | **★** | **★** | **★★** | **-** | **★★** | **★** | 8 |
| Vitery (2020) | **★** | **★** | **-** | **★★** | **★★** | **★★** | **★** | 9 |
| Haapala (2021) | **★** | **-** | **-** | **★★** | **★** | **★★** | **★** | 7 |
| de Fruiter (2021) | **★** | **-** | **-** | **★★** | **-** | **★★** | **★** | 6 |
| Thillan (2021) | **★** | **★** | **-** | **★★** | **★★** | **★★** | **★** | 9 |
| Francis (2021) | **★** | **★** | **-** | **★★** | **★★** | **★★** | **★** | 9 |

Two reviewers evaluated study quality independently using the Newcastle - Ottawa Quality Assessment Scale adapted for cross-sectional studies. Abbreviations: REP =Representative of the sample. SAM = Sample size. NREP = Non-respondents. AE= Ascertainment of the exposure (risk factor). COM= The subjects in different outcome groups are comparable ASS= Assessment of the outcome. STAT =Statistical tests. Seven or more stars were considered as High quality and less than seven as poor quality.
